# A Portrait of Current Radiation Oncology Twitter Influencers

**DOI:** 10.7759/cureus.10838

**Published:** 2020-10-07

**Authors:** Sharifa Beroual, Chirag Shah, Miriam Knoll, Houda Bahig, Carole Lambert, Daniel Taussky

**Affiliations:** 1 Radiation Oncology, Centre Hospitalier de l'Université de Montréal, Montreal, CAN; 2 Radiation Oncology, John Theurer Cancer Center at Hackensack University, Hackensack, USA; 3 Radiation Oncology, Centre Hospitalier de l'Université de Montréal - Hôpital Notre-Dame, Montreal, CAN

**Keywords:** social media analytics, radiation oncology, influencers, twitter, academia

## Abstract

Introduction

We aimed to characterize the most influential radiation oncologists on Twitter, the correlation between their Twitter activity and their academic profiles as measured by the Scopus H-index as well as their activity around the American Society for Radiation Oncologists (ASTRO) 2018 meeting.

Methods

We defined radiation oncology influencers as any radiation oncologist with 500 or more followers on Twitter through the first two weeks of August 2019. We collected their available characteristics, their Scopus H-index, and Twitter metrics. We examined their general Twitter activity as well as their specific activity before, during, and after the 2018 ASTRO annual meeting. We identified the most frequent tweet content categories for each influencer.

Results

We identified 48 radiation oncologist influencers; 79% were male, 75% were based in the United States, and 94% were affiliated with an academic center. Among them, 44% had high H-indices of ≥21, an average value in academic faculty for full professors or department heads.

There were no correlations between H-index and Twitter metrics such as the number of individuals the influencer was following (p = 0.58), the number of followers (p = 0.66), the number of tweets (p = 0.88), and the number of likes (p = 0.54). During the period around ASTRO 2018, the mean number of tweets per influencer was 4437 (range 87-93,000).

Conclusion

Current radiation oncology influencers are predominantly North American males from academic institutions. A correlation between academic productivity as measured by the H-index and Twitter metrics was not demonstrated. The fact that some influencers had a low H-index supports that a high academic profile as measured by traditional metrics is not necessary to have a voice in the Twitter radiation oncology community.

## Introduction

Influencers in social media are defined as people who have built a reputation for their knowledge and expertise on a particular topic and who have a significant footprint in their community [1]. They may use their gained visibility for different reasons such as to communicate with colleagues, educate the public, or contribute to marketing efforts. Influencers can shape audience attitudes through blogs, tweets, and the use of other social media platforms [2]. The use of social media has expanded in medicine in the past few years [3], especially Twitter, which has proven to be a growing platform for different specialties allowing instant information sharing, continuous medical education, research collaboration opportunities, networking, and advocacy [4,5]. This expanding importance has taken hold of radiation oncology, evidenced by the initiative of selecting eight radiation oncologists designated as influencers at the 2018 annual ASTRO meeting (https://twitter.com/astro_org/status/1049726528018374656) to be followed on Twitter. This is representative of an official recognition of this platform for data sharing and dissemination of relevant information.

Nevertheless, we know very little of who the current Twitter influencers in radiation oncology are and what is their disseminated content. As radiation oncologists, we need to know who is influencing us to detect possible bias such as origin, sex, or obvious financial bias of influencers. Furthermore, we examined the academic profiles of the most influential radiation oncologists on Twitter to know whether being an influencer is associated with being an often-cited scientist. Conferences are potentially a time of increased Twitter activity to promote research and discussion. We analyzed Twitter activity around an important radiation oncology conference.

## Materials and methods

Identifying radiation oncology influencers on Twitter

The main objective of this study was to analyze individual radiation oncologists that reach a large audience through Twitter. Because these data are available to everyone on Twitter, no ethics committee permission was demanded. Therefore, we did not include institutional accounts as they are managed by several people and not necessarily radiation oncologists. We realized early on in our research that it would be unrealistically time-consuming to manually gather all tweets for analysis. After identifying the seemingly topmost popular Twitter accounts, we decided to establish an arbitrary cutoff of 500 followers in order to have a sufficient number of accounts to analyze. We ended up with a total of 48 Twitter influencers. We identified influencers in radiation oncology based on having greater than 500 followers. Radiation oncologists were searched on Twitter using the search tool on Twitter, by typing in the following keywords: “radonc,” “radiation oncologist,” and “radiation.” 

Twitter metrics were collected on the physician’s profile including gender (based on profile picture and name), continent, and country in which the individual practices, hospital or practice affiliations, account creation date, number of followers, and number of individuals he or she was following.

Data collection and analysis

We chose to analyze data regarding Twitter activity during the 2018 ASTRO annual meeting that took place between October 21 and 24 of 2018. We manually searched Twitter for self-identified radiation oncologists (#radonc, radiation oncologist, and radiation). All tweets were analyzed individually by the first author in August 2019. We were more specifically looking at the Twitter account activity (a) before October 1 to 20, (b) during October 21 to 24, and (c) after October 25 to November 1, the ASTRO meeting. The number of tweets were categorized as either (1) original tweets by the influencer, (2) quoted from another tweet with edit, or (3) retweets (i.e., tweet from another user without any editing). Additionally, we analyzed the number of comments, likes, and retweets.

Furthermore, we identified the content of the tweets. Thirteen different categories were identified according to the most frequent types of tweets and the main message conveyed by the tweet, as utilized by Ciprut et al. [4]. These included advocacy (tweets that support or recommend a particular cause or policy, e.g., ways to help protect patients from unnecessary delays in care), guidelines (clinical practice lines, e.g., ASTRO guidelines on hypofractionated radiotherapy for localized prostate cancer), awards (acknowledgments on a contribution/accomplishment, e.g., award from ASTRO recognizing a physician's collaboration between thoracic surgery and radiation oncology), self-promotion (individual posting content promoting or publicizing themselves, including awards/publications/research contribution, etc.), conference (e.g., ASTRO annual meeting conference content such as slides from a presentation), education (content that is instructive/informative, e.g., what to know about proton therapy - intended for patients or how to deliver bad news to your patients), research (studies that are not published, e.g., preclinical work underway in animal models using proton therapy or flash technology), publication (work that is published in an academic journal, book, or thesis), industry (imaging technologies and manufacturers), professional societies (such as ASTRO), patients (content that is related to patients, e.g., cancer survivors posting about their journey), and miscellaneous (content that does not belong to any of the other categories). Tweets were classified into only one category; tweets that could be classified into more than one category were classified into the category judged to be the most representative. Analysis and archiving were done by creating an excel sheet that contained these 13 different categories. The accounts of 48 influencers were individually analyzed (during the 2018th ASTRO annual meeting period) by manually scrolling the Twitter page and categorizing every tweet.

To assess the influencer’s academic productivity, we initially searched on Google Scholar for their H-index [6], which was compared to the number of followers and number of tweets. As some influencers did not have an H-index on the Google Scholar platform, we ultimately used the Scopus H-index exclusively as it contained the H-index of all the influencers. Academic institutions were defined as either university hospitals or as hospitals affiliated with a university.

Summary statistics included mean, standard deviation, median, range, and frequency of influencers’ characteristics and Twitter content. Pearson correlation was used to correlate the academic productivity (as measured by the H-index) with various Twitter metrics. A generalized linear mixed model was used to analyze the variation in Twitter activity across time; using Bonferroni correction, a p value below 0.003 was considered statistically significant.

## Results

Table 1 shows characteristics of the top 48 radiation oncology influencers. The median, interquartile range (IQR) number of followers, accounts followed, and number of tweets were 1517 (1172-2309), 616 (297-1045), and 1365 (651-3866), respectively. Ten (21%) were female radiation oncologists. 75% of the account holders were from the United States. All but three (6%) radiation oncologists were working in an academic center. A total of 29% (14/48) were from three universities only: Cleveland Clinic, MD Anderson Cancer Center, and Northwestern University, which accounted for 11%, 13%, and 6% of the accounts, respectively. 

**Table 1:** Descriptive statistics of top Twitter influencers in radiation oncology (n = 48) IQR: Interquartile range.

Descriptive statistics		
Gender	Number	%
Male	38	79
Female	10	21
Continent		
Australia	5	10
Europe	4	8
North America	39	81
Country		
Australia	5	10
Europe excluding UK	2	4
UK	2	4
Canada	3	6
USA	36	75
Affiliations		
Cleveland Clinic	5	11
MD Anderson Cancer Center	6	13
Northwestern University	3	6
Others	34	71
	Median	IQR
Number of followers	1517	1172-2309
Accounts followed	616	297-1045
Number of tweets	1365	651-3866
Number of likes	2824	943-6602
Scopus H-index	15	8.3-37.3

Table 2 illustrates the results of the content analysis around the 2018 annual ASTRO meeting (October 1 to November 1, 2018). The majority of tweets were conference-related (mean 9.6), followed by publications (mean 7.3), advocacy (mean 5.7), and education-related (mean 5.6). 

**Table 2:** Overall distribution of tweets by category from October 1 to November 1, 2018 (before/during/after the 2018 annual ASTRO meeting)

Category of tweet	Mean	Range
Advocacy	5.7	0-52
Guidelines	0.3	0-2
Award	2.1	0-16
Self-promotion	1.4	0.12
Media appearance	0.7	0-8
Conference	9.6	0-42
Education	5.6	0-33
Publication	7.3	0-48
Research	3.9	0-27
Industry	0.02	0-1
Professional societies	1.8	0-15
Patients	0.7	0-8
Miscellaneous	3.5	0-17

A total of four influencers (8%) had an H-index of ≤3. Indices associated with senior faculty was seen in an important minority of radiation oncologists: 21 (44%) had an H-index of ≥21, while 15 (31%) had an H-index of ≥31. 

Table 3 demonstrates the correlations between Twitter metrics and Scopus H-index. Overall there were no correlations between Twitter metrics such as the number of followers (p = 0.66), the number of tweets (p = 0.88), the number of likes (p = 0.54) or the number of accounts the influencer was following (p = 0.58), and the H-index. However, during the 2018 ASTRO annual meeting, award-related content was positively related with H-index (r = 0.4, p = 0.006), while education-related content was inversely correlated (r = -0.32, p = 0.03).

**Table 3:** Correlations between Scopus H-index and Twitter metrics

Overall metrics	Pearson correlation coefficient	p value
Number of followings	-.08	.6
Number of followers	-.07	.7
Number of tweets	.02	.9
Number of likes	-.09	.5
Tweet content		
Advocacy	-.04	.8
Guidelines	-.1	.5
Award	.4	.006
Self-promotion	-.11	.5
Media appearance	.21	.2
Conference	-.05	.8
Education	-.32	.03
Publication	-.11	.5
Research	-.18	.2
Industry	.05	.7
Professional societies	.08	.6
Patients	.09	.6
Miscellaneous	-.11	.5

When looking at tweet content, Table 4 shows variation in Twitter activity around the 2018 ASTRO Annual meeting, with a statistically significant increase in Twitter activity before and during the conference regarding the number of retweets, award, conference, and publication-related content versus after the annual meeting. 

**Table 4:** Content and activity tweet changes before, during, and after the 2018 ASTRO annual meeting categorized according to criteria by Ciprut et al. [4] ASTRO: American Society for Radiation Oncologists.

	Pre-ASTRO		Per-ASTRO		Post-ASTRO		
	Mean	SD	Mean	SD	Mean	SD	p value
Original tweets	7.23	9.59	7.77	11.35	4.38	5.94	0.06
Retweets	9.08	12.45	8.63	12.77	3.75	5.11	0.001
Advocacy	2.38	4.51	1.75	4.96	1.6	3.26	0.50
Guidelines	0.15	0.41	0.1	0.309	0	0	0.05
Award	0.98	1.94	0.98	1.59	0.19	0.49	0.003
Self-promotion	0.44	0.92	0.54	1.17	0.38	0.89	0.6
Media appearance	0.4	0.89	0.13	0.73	0.17	0.47	0.1
Conference	3.15	4.4	5.48	7.2	1.02	1.64	0.0001
Education	2.4	4.11	1.77	2.84	1.42	1.9	0.2
Publication	2.19	3.2	3.9	5.15	1.21	1.58	0.0001
Research	1.85	3.7	1.42	2.22	0.65	1.82	0.04
Industry	0.02	0.122	0	0	0	0	0.3
Professional society	0.96	2.08	0.33	0.694	0.5	1.22	0.02
Patients	0.44	1.009	0.08	0.279	0.17	0.559	0.008
Miscellaneous	1.48	2.36	0.56	1.128	1.42	2.08	0.007

## Discussion

Overall, the results show that the top radiation oncologist influencers are mainly (79%) male from the United States. This is representative of the proportion of male members (75%) of ASTRO (www.astro.org/membership). Men are therefore not necessarily overrepresented in this group. The majority of Twitter influencers were from well-established academic institutions, with only three influencers being from non-academic centers. This suggests a high representation of academic institutions on Twitter compared to an ASTRO survey distributed in 2017 in the United States, which found that a minority (41%) of responders were working in academic institutions. Up to 44% of the identified Twitter influencers had H-indices of ≥21, an average value in academic faculty for full professors or department heads in radiation oncology and considered representative of a mature academic position [6,7], while 31% had H-indices of ≥31, associated with senior faculty as seen in a systemic review of the recent medical literature [8]. On the other hand, a minority had low academic productivity, with only 8% having an H-index of ≤3. In a similar report looking at influencers in plastic surgery, the authors characterized the top 100 social media influencers using the topic search "plastic surgery" in July 2017 using Right Relevance software (Sunnyvale, California). As in our study, there was a very similar geographic distribution with 77% of the influencers being from North America compared to the 81% in our study [2]. While it can be expected that a majority of Twitter users would be from the United States given the origin of the application, this geographical distribution may introduce a bias in the discussed content and disseminated information toward North American realities. 

This study provides content analysis of influencers' tweets within the field of radiation oncology. The majority of tweets were related to academic content. However, Twitter metrics were not correlated to the Scopus H-index. Previous studies analyzing the use of Twitter during medical conferences in other medical specialties have demonstrated that physician influencers may be important drivers of conference participation [9,10]. In a study by the Cardiac Society of Australia & New Zealand evaluating the reach and impact of Twitter in scientific meetings, an average of nine tweets per hour and six tweets per participant during their 2013 61st Annual Scientific Meeting were reported [11]. Another study by the Spanish Association of Surgeons analyzed Twitter activity during their Annual Scientific Meeting from 2013 to 2016 and found that the rate of tweets from influencers was 65% in 2013 and decreased to 35% in subsequent years [12], pointing to a more generalized use of Twitter over time. Although speculative, it is possible that Twitter plays an increasingly stronger academic role as an increasing number of users engage. As multidisciplinary tumor boards via social media such as Twitter have already become reality [13,14], Twitter has the potential to become a space for multidisciplinary exchanges where patients, health professionals, and researchers can engage more easily. It could also give a stronger voice to the role of radiotherapy in oncological care by reaching and educating the population directly.

We arbitrarily chose Twitter influencers as being those who had more than 500 followers to include about 50 of the most influential Twitter members of the radiation oncology field. This list notably included all the influencers that the 2018 ASTRO meeting recommended to follow during the conference. To classify the content of the tweets, we used the methodology described by Ciprut et al. in urology [4], which, in our opinion, can be translated to any medical specialty. Given that the tweets were all categorized manually, this is certainly a limitation of the method that can be subject to a certain level of subjectivity. Additionally, even though there could be some overlap, each tweet was classified in only one category, the felt to be the most representative. 

## Conclusions

In conclusion, we found that current radiation oncology influencers are predominantly North American males from academic institutions. A correlation between academic productivity as measured by the H-index and Twitter metrics was not demonstrated. The fact that some influencers had a low H-index supports that a high academic profile as measured by traditional metrics is not necessary to have a voice in the Twitter radiation oncology community. The impact of Twitter for the propagation of opinions, representation of our specialty, education of our patients, and the need for quality control are topics that will require further attention as Twitter use continues to grow in our field.
